# Efficacy of Postoperative Unilateral Neck Irradiation in Patients with Buccal Mucosa Squamous Carcinoma with Extranodal Extension: A Propensity Score Analysis

**DOI:** 10.3390/cancers13235997

**Published:** 2021-11-29

**Authors:** Chia-Hsin Lin, Chien-Yu Lin, Kang-Hsing Fan, Sheng-Ping Hung, Yung-Chih Chou, Chia-Jen Liu, Wen-Chi Chou, Yen-Chao Chen, Shiang-Fu Huang, Chung-Jan Kang, Kai-Ping Chang, Hung-Ming Wang, Ann-Joy Cheng, Joseph Tung-Chieh Chang

**Affiliations:** 1 Department of Radiation Oncology, Linkou Chang Gung Memorial Hospital, Chang Gung University, Taoyuan 333, Taiwan; chlin1016@cgmh.org.tw (C.-H.L.); qqvirus@cgmh.org.tw (C.-Y.L.); kanghsing@cgmh.org.tw (K.-H.F.); sphung89@cgmh.org.tw (S.-P.H.); russellhome@cgmh.org.tw (Y.-C.C.); annjoycheng@gap.cgu.edu.tw (A.-J.C.); 2 Division of Hematology and Oncology, Department of Medicine, Taipei Veterans General Hospital, Taipei 112, Taiwan; cjliu5@vghtpe.gov.tw; 3 Institute of Public Health, National Yang-Ming University, Taipei 112, Taiwan; 4 Department of Medical Oncology, Chang Gung Memorial Hospital at LinKou, Chang Gung University, Taoyuan 333, Taiwan; f12986@cgmh.org.tw (W.-C.C.); whm526@cgmh.org.tw (H.-M.W.); 5 Department of Radiation Oncology, Chang Gung Memorial Hospital-Keelung, Keelung 204, Taiwan; eric0705@cgmh.org.tw; 6 Department of Otorhinolaryngology, Linkou Chang Gung Memorial Hospital, Chang Gung University, Taoyuan 333, Taiwan; bigmac@cgmh.org.tw (S.-F.H.); keny@cgmh.org.tw (C.-J.K.); changkp@cgmh.org.tw (K.-P.C.); 7 Department of Medical Biotechnology and Laboratory Science, College of Medicine, Chang Gung University, Taoyuan 333, Taiwan

**Keywords:** postoperative radiotherapy, unilateral radiotherapy, buccal mucosa squamous cell carcinoma, extranodal extension, propensity score analysis

## Abstract

**Simple Summary:**

The application of unilateral neck irradiation may be able to reduce the toxicities associated with postoperative radiotherapy. However, the feasibility of using unilateral neck irradiation in patients with buccal mucosa squamous cell carcinoma (BMSCC) with extranodal extension (ENE) remains unexplored. The aim of our retrospective study was to evaluate whether unilateral irradiation is safe for ENE+ patients with well-lateralized BMSCC. We demonstrated in propensity-matched cohorts (123 patients) the comparability of clinical outcomes in patients treated with postoperative unilateral versus bilateral radiotherapy. We identified the number of ENEs ≥ 4 as a potent risk factor for contralateral nodal recurrence and established a prognostic model accordingly. This study shall justify the use of unilateral neck irradiation and help physicians optimize radiation fields.

**Abstract:**

Unilateral radiotherapy (RT) as a postoperative treatment for multiple ipsilateral lymph node (LN) metastases remains controversial. We investigated the efficacy of postoperative unilateral RT for buccal mucosa squamous cell carcinoma (BMSCC) with extranodal extensions (ENEs). We retrospectively reviewed the clinical records of 186 patients with ENE+ BMSCC who received postoperative RT during 1997–2016. Propensity score matching was used to establish comparable cohorts. The endpoints were contralateral nodal control (CLNC), overall survival (OS), disease-free survival (DFS), distant metastasis-free survival (DMFS), local control (LC), and regional control (RC). After matching, 123 patients were selected for analysis; 45 (36.6%) and 78 (63.4%) patients underwent unilateral and bilateral RT, respectively. The median follow-up was 36.27 months. The survival outcomes in the unilateral and bilateral RT groups were similar: 3-year CLNC (85.6% vs. 89.1%, *p* = 0.748), OS (53.2% vs. 57.4%, *p* = 0.229), DFS (46.5% vs. 48.6%, *p* = 0.515), DMFS (70.7% vs. 72.0%, *p* = 0.499), LC (78.0% vs. 75.6%, *p* = 0.692), and RC (79.9% vs. 76.2%, *p* = 0.465). On multivariable Cox regression analysis, unilateral and bilateral RT showed comparable outcomes; the number of ENEs ≥ 4 was the only significant prognostic factor for all clinical outcomes. Using decision tree analysis, we classified our patients to have a low, intermediate, or high risk of contralateral failure based on three factors: number of ENEs, margin status, and tumor stage. In conclusion, postoperative unilateral RT did not worsen outcomes in patients with ENE+ BMSCC in this cohort. The decision tree model may assist physicians in optimizing and tailoring radiation fields.

## 1. Introduction

Head and neck squamous cell carcinoma (HNSCC) has a high propensity to spread to cervical lymph nodes (LNs) due to abundant lymphatic networks in the upper aerodigestive tract. Furthermore, compelling evidence has shown that cervical LN metastasis portends a poor prognosis in patients with HNSCC [1,2]. In this regard, a conventional paradigm of radiotherapy (RT) for advanced stage HNSCC often involves the bilateral neck (bilateral RT) to eradicate potential metastatic foci in the cervical LNs, as reported by Lindberg et al. [3]. However, RT could contribute to acute and long-term toxicities, and reducing RT volume, such as that in unilateral RT, has been associated with reduced toxicities and improved quality of life [4,5,6]. Emerging studies further suggested that unilateral RT can aid in achieving excellent disease control and survival similar to that with bilateral RT in some well-selected and well-lateralized oral and oropharyngeal cancers [6,7,8,9,10,11,12,13,14,15,16,17,18,19,20,21,22,23,24]. Additionally, with increased use of advanced imaging modalities such as contrast-enhanced computed tomography, magnetic resonance imaging (MRI), and positron emission tomography (PET/CT), and sentinel LN biopsy, both nodal and tumor staging has become more accurate, which raises concerns about the necessity of elective bilateral neck RT.

Extranodal extension (ENE) has been well recognized as one of the most significant risk factors for disease control and survival in patients with HNSCC [25,26]. Postoperative chemoradiotherapy is regarded as the standard treatment for patients with ENE [27,28]. In this high-risk patient population, significant controversy remains about the use of unilateral RT, given that ENE seemed to correlate with treatment failures, including contralateral LN recurrence [22,23]. Nevertheless, the prognosis of patients with ENE may not be homogenously dismal [29]. Additionally, the impact of unilateral RT on contralateral LN control in ENE-positive (ENE+) patients was unclear. Contralateral LN failure rates of 0%–2.8% were reported in prior studies despite the presence of ENE in the majority of the subjects after unilateral RT [21,24]. However, there is currently no published study investigating and directly comparing the clinical outcomes of unilateral RT with those of bilateral RT in ENE+ HNSCC patients.

In Taiwan, an endemic betel quid chewing area, buccal mucosa squamous cell carcinoma (BMSCC), represents a common type of malignancy [30], accounting for 30–40% of oral cavity cancers. The lateralization of BMSCC has justified the use of unilateral RT in postoperative treatment; however, the use of unilateral RT is controversial for diseases with multiple ipsilateral LN metastases [22,31]. This study aimed to evaluate further whether unilateral irradiation is feasible for ENE+ patients with well-lateralized BMSCC.

## 2. Results

### 2.1. Patient and Treatment Characteristics

**Figure 1:**
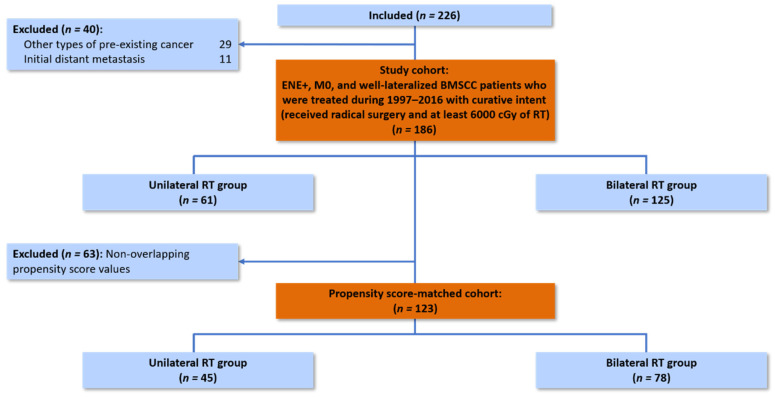
Eligible patients undergoing propensity-score matching.

In total, 186 patients at our institution were enrolled in the study following the predetermined inclusion and exclusion criteria, with 61 (32.8%) patients treated with unilateral RT and 125 (67.2%) patients treated with bilateral RT (Figure 1). The baseline characteristics of patients are listed in Table 1. Notably, the unilateral RT group had higher rates of lymphatic invasion (21.3% vs. 9.6%; *p* = 0.028) and lower rates of soft tissue invasion (75.4% vs. 88.0%; *p* = 0.028). After PSM, 45 (36.6%) and 78 (63.4%) patients were treated with unilateral and bilateral RT, respectively. All clinicopathological factors were well balanced between the matched subgroups, except for the types of RT techniques (Table 1). Moreover, the ipsilateral irradiated volumes were balanced; the level I-V LNs plus supraclavicular fossa were covered in most of the study patients (75.6%) among both groups (Supplementary Table S1). All subsequent analyses were performed using the propensity-matched cohort.

### 2.2. Survival Analyses

**Figure 2:**
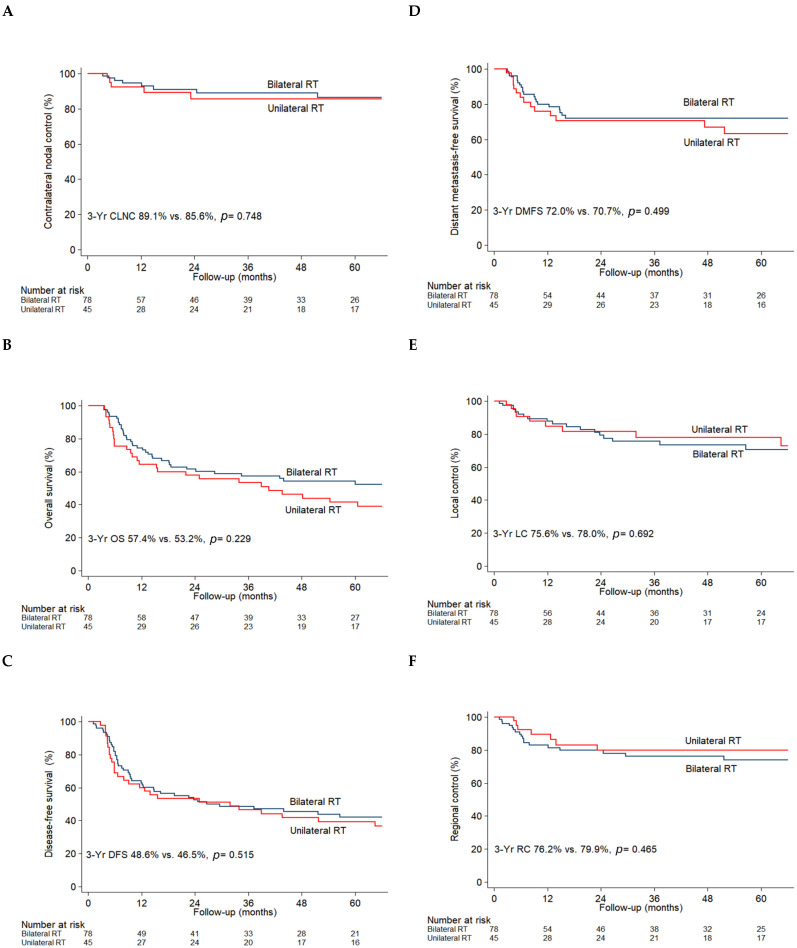
The Kaplan–Meier estimates of (**A**) contralateral nodal control (CLNC), (**B**) overall survival (OS), (**C**) disease-free survival (DFS), (**D**) distant metastasis-free survival (DMFS), (**E**) local control (LC), and (**F**) regional control (RC) for patients treated with bilateral radiotherapy (RT) (blue line) versus unilateral radiotherapy (RT) (red line) in the propensity-matched cohort.

The median follow-up time was 36.27 months (range, 3.55–218.45 months) for the entire propensity-matched cohort (*n* = 123). Among them, 71 (57.7%) expired, 35 (28.5%) had distant failure, 25 (20.3%) had regional failure, and 30 (24.4%) had local failure at the time of the last follow-up. The failure patterns are shown in Supplementary Figure S1. Kaplan–Meier estimates revealed that unilateral RT yielded similar clinical outcomes as those obtained with bilateral RT (CLNC rate at 3 years: 85.6% vs. 89.1%, *p* = 0.748; OS rate at 3 years: 53.2% vs. 57.4%, *p* = 0.229; DFS rate at 3 years: 46.5% vs. 48.6%, *p* = 0.515; DMFS rate at 3 years: 70.7% vs. 72.0%, *p* = 0.499; LC rate at 3 years: 78.0% vs. 75.6%, *p* = 0.692; and RC rate at 3 years: 79.9% vs. 76.2%, *p* = 0.465; Figure 2A–F). Consistent findings were observed when the analysis was repeated after the exclusion of patients not receiving adjuvant cisplatin-based concurrent chemoradiotherapy (Supplementary Figure S2).

### 2.3. Evaluation of Prognostic Factors

Univariate analyses of clinical outcomes by RT laterality and prognostic and confounding factors are shown in Supplementary Table S1. All significant univariate factors, types of RT techniques, and RT laterality were included in the multivariate Cox regression models (Table 2). In both univariate and multivariate analyses, unilateral RT did not result in worse outcomes compared with those in bilateral RT. ENE number ≥ 4 nodes was the only independent risk factor remaining significant in all clinical outcomes. Pathologic tumor stage 3–4 was associated with unfavorable outcomes, including OS, DFS, DMFS, and RC. Lymphatic invasion was correlated with poorer OS and DFS, while the surgery to RT interval of more than six weeks was related to inferior LC.

### 2.4. Prognostic Model for CLNC

**Figure 3:**
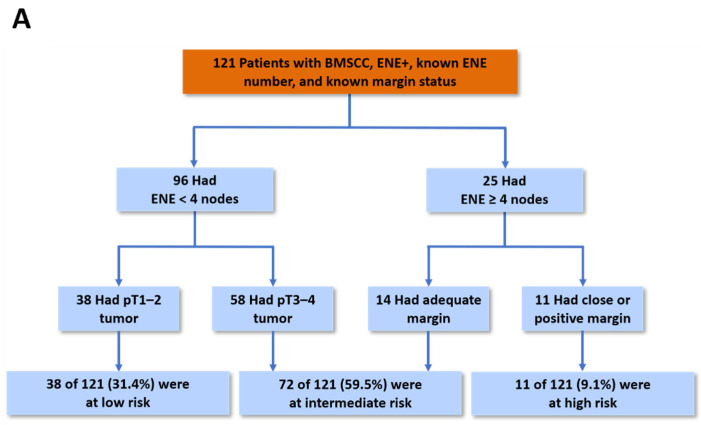

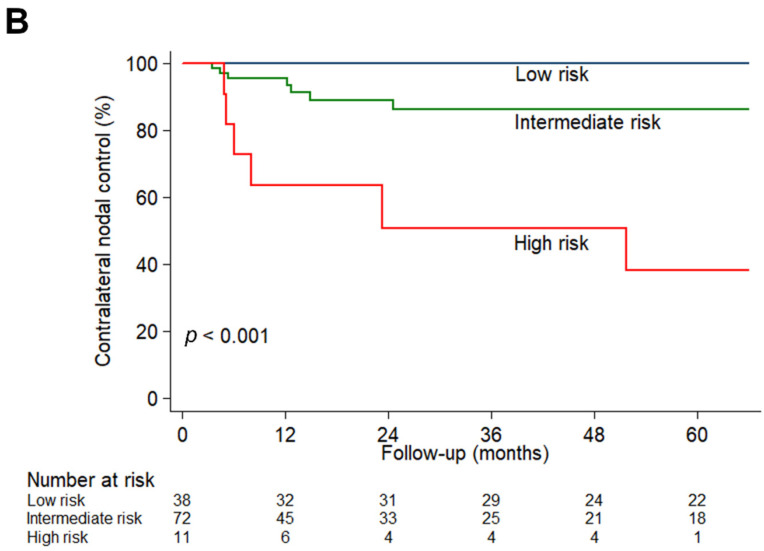
Classification of the study patients into risk-of-contralateral-failure groups and Kaplan–Meier estimates of contralateral nodal control according to those categories in the propensity-matched cohort. Decision tree analysis was applied to identify prognostic factors with the most influential predictive importance in a model of contralateral nodal control and to divide patients into groups of low, intermediate, and high risk of contralateral nodal failure. The prognostic factors in the model were number of extranodal extension (ENE), margin status, and tumor stage. (**A**) shows the resulting classifications. (**B**) shows contralateral nodal control in the classified patients. The 3-year rates of contralateral nodal control were 100.0%, 86.2%, and 50.9% in the low-risk, intermediate-risk, and high-risk groups, respectively.

We subsequently developed a prognostic model for estimating CLNC among BMSCC patients. Based on the decision tree analysis results, the number of ENEs was the major prognostic factor (Chi-square = 10.09, *p* = 0.004), followed by margin status (Chi-square = 6.87, *p* = 0.009) and tumor stage (Chi-square = 4.23, *p* = 0.040) (Figure 3A). In order to obtain a more clinically useful tool, groups with similar risk for contralateral nodal failure were further refined into three distinct groups: high-risk group (ENE number ≥ 4 with close/positive margins), intermediate-risk group (ENE number ≥ 4 with an adequate margin or ENE number < 4 with a pT3–4 tumor), and low-risk group (ENE number < 4 with pT1–2 tumor). Among 121 eligible patients (data on ENE number were missing for two patients), the prognostic model was significantly associated with the 3-year CLNC rates in the high-, intermediate-, and low-risk groups, which were 50.9%, 86.2%, and 100.0%, respectively (Figure 3B). Numerically, when compared with bilateral RT, unilateral RT seemed to yield similar 3-year CLNC rates in the low-risk (100.0% vs. 100.0%) and intermediate-risk (85.2% vs. 86.4%) groups, while it appeared to yield a worse 3-year CLNC rate in the high-risk group (57.1% vs. 37.5%) (Supplementary Figure S3). However, the number of events was too small.

### 2.5. Contralateral Nodal Failure

Among all patients with cNF (*n* = 13: five, unilateral neck RT and eight, bilateral neck RT), only two patients (15.4%) had an isolated cNF and were successfully salvaged by means of neck dissection followed by postoperative RT. Nine patients with cNF also had distant metastasis; thus, neck salvage treatment was deferred. Of the two patients with cNF and simultaneous failure at the primary site, one patient underwent surgery and postoperative RT plus chemotherapy but died of treatment-related infection, while the other patient did not receive salvage treatment due to poor general condition and died of severe tumor bleeding. The characteristics of patients with cNF are shown in Table 3.

### 2.6. Complications

We did not find any significant difference between patients receiving bilateral RT and unilateral RT on both acute and late radiation-induced complications (Table 4). Any grade 3 or worse acute toxicity accounted for 51.4% and 43.6% (bilateral versus unilateral RT), respectively. Any grade 2 or worse late toxicity accounted for 29.0% and 32.1% (bilateral versus unilateral RT), respectively. No grade 5 complication was noted.

## 3. Discussion

Regarding elective nodal irradiation for patients with HNSCC, the RT volume chosen is based on the balance between the risk of recurrence and treatment-related toxicities. ENE is one of the most important prognostic factors in patients with HNSCC, and CCRT was shown to improve tumor control and survival [27,28]. To the best of our knowledge, this is the first study utilizing propensity score analysis for the direct comparison of clinical outcomes in patients with pathologically confirmed ENE+ BMSCC treated with postoperative unilateral or bilateral RT. In our study, unilateral RT led to oncologic outcomes comparable to those of bilateral RT with respect to all study endpoints. We observed an acceptable 3-year CLNC rate of 85.6% for those treated with unilateral RT, which was not significantly different from that for bilateral RT. Importantly, only two patients developed isolated contralateral nodal failure, while most cases of contralateral nodal failure (69.2%) occurred simultaneously or were preceded by distant failure, suggesting the limited value of adding RT to the contralateral neck. It highlighted the importance of early detection of distant metastasis as well as the investigation of effective systemic treatment for this high-risk patient subgroup in further clinical trials.

Previous studies on the omission of contralateral neck RT have focused primarily on early-stage oral or oropharyngeal tumors without complete data of ENE status or with limited patients with ENE+ disease [7,8,9,10,11,12,13,14,15,16,17,18,19] (Supplementary Table S2). Modern studies have evaluated the influence of ENE but reported conflicting results in terms of contralateral nodal failure incidence [6,20,21,22,23,24]. Vergeer et al. (*n* = 123) and Lynch et al. (*n* = 136) reported a higher chance of contralateral nodal failure (15–20%) among patients with ENE than among those without ENE (2–9%) [22,23]. However, in a recent prospective phase II study (*n* = 72), Contreras et al. showed that only 2.8% (*n* = 2) of patients developed failure at the unirradiated neck despite the presence of ENE in 64% of their cohort [24]. Chin et al. (*n* = 48) also reported no contralateral nodal failure with the use of unilateral RT despite the presence of ENE+ disease in a significant proportion (77%) of their participants [6].

This discrepancy might be attributed to the heterogeneity in tumor subsites, failure pattern, and survival rates of patients with ENE+ disease [26,29]. In a large retrospective study of patients with pN3b oral cavity SCC (*n* = 365), Liao et al. reported that the number of ENEs was an independent adverse prognostic factor for regional recurrence, distant metastasis, and survival, and this was further incorporated for risk stratification in patients with pN3b disease [29]. This was consistent with our findings in patients with ENE+ BMSCC. We further identified that ENE number ≥ 4 was an independent risk factor for contralateral nodal failure in the current study. The 3-year incidence rate of contralateral nodal failure was 8% in patients with ENE number < 4, compared to a rate of 34% in patients with an ENE number of ≥4 (*p* < 0.001). However, most of the published cohorts did not report detailed information on the number of ENEs, and thus, further detailed analysis could not be performed [6,7,8,9,10,11,12,13,14,15,16,17,18,19,20,21,22,23,24,32].

Accordingly, we believe it would be prudent to recommend that all patients with ENE+ BMSCC should be safely treated with unilateral RT considering the varying prognoses of patients with ENE+ disease. Although none of the putative prognostic factors other than the number of ENEs was significant for the risk of CLNC, margin status and pathological tumor stage were also defined as risk factors using the tree decision analysis. Based on this, a novel prognostic model was established that identified three distinct risk categories for contralateral nodal failure. Furthermore, the proposed prognostic model was shown to retain its discriminative ability in both unilateral and bilateral RT subgroup analyses. If independently validated, this prognostic stratification model may assist physicians in optimizing and tailoring radiation fields to cater to the individual patient’s risk (e.g., unilateral RT for the low-to-intermediate-risk group; bilateral RT for the high-risk group) in patients with ENE+ BMSCC.

However, it should be noted that a large proportion of contralateral nodal failures involved concomitant or antecedent distant recurrences in patients with ENE. This suggested that adjuvant systemic treatment may also be considered instead of merely applying elective contralateral neck RT. After all, it has been reported that bilateral neck RT led to more acute and late complications and had a negative impact on patients’ quality of life compared with unilateral neck RT [6,33].

As this was a retrospective study, there are several inherent limitations. First, although the two treatment arms were well balanced in terms of multiple clinicopathological variables using the PSM method, we could not rule out the possibility that some unknown confounding factors were imbalanced and masked potential differences in the efficacy between bilateral RT and unilateral RT. For example, we matched our patients based on the number of ENEs, but we could not analyze the size of ENEs, which can potentially affect prognosis in patients with HNSCC [34,35]. Importantly, the use of PSM unavoidably resulted in a smaller sample size, which compromised the statistical power of the current study to detect the true effect. Moreover, this study spanned a long observation period of 19 years, during which RT techniques greatly varied. More specifically, we found that there was a relatively larger proportion of patients treated with conventional RT techniques in the unilateral RT arm (14/61, 23.0%) versus the bilateral RT arm (5/125, 4%). Although we did not incorporate the types of RT techniques into factors for PSM, we carried out the multivariate cox regression analysis to eliminate this imbalance, showing consistent findings with respect to the equivalent outcomes of unilateral RT versus bilateral RT arm. Finally, there was a great risk of information bias with respect to the collection of data on toxicity outcomes in a retrospective study, which may explain why unilateral RT did not appear to have a more favorable toxicity profile in the current study. Certainly, a prospective study design is needed to confirm our findings.

## 4. Materials and Methods

### 4.1. Patients

In this study, we enrolled newly diagnosed buccal mucosa (cheek) squamous cell carcinoma patients who underwent definitive treatment with radical surgery and postoperative RT between January 1997 and December 2016 from the prospectively acquired database in the cancer registry of our center. The inclusion criteria were as follows: histopathologically confirmed BMSCC and ENE, well-lateralized tumors, no evidence of contralateral cervical lymphadenopathy, radical surgery and postoperative RT as the primary treatment, and a prescribed radiation dose ≥6000 cGy. The exclusion criteria included the presence of distant metastasis at diagnosis and the presence of a second primary cancer before surgery.

The study was conducted according to the guidelines of the Declaration of Helsinki and was approved by Chang Gung Medical Foundation Institutional Review Board (IRB No. 201900147B). The date of approval is 30 January 2019. Patient consent was waived due to the IRB reviewed and determined that it is expedited review according to case research or cases treated or diagnosed by clinical routines.

All the patients underwent pretreatment examinations and staging workup, which included medical history-taking, complete physical examination, flexible fiberoptic nasopharyngoscopy, complete blood count, liver/renal function tests, CT or MRI of the head and neck region, chest X-ray, bone scan, and liver ultrasonography. Presurgical PET/CT scans were routinely obtained for most of the patients after 2005. Clinical staging was based on the American Joint Committee on Cancer (AJCC), 8th edition [36].

### 4.2. Treatment

Surgical removal of the primary tumor was performed with adequate margins. Modified radical neck dissection (level I–V) was performed in patients with clinically or radiographically detected metastatic LNs, whereas supraomohyoid neck dissection (levels I–III) was applied in clinically or radiographically LN-negative patients. Postoperative concurrent chemoradiation (CCRT, 60–66 Gy) was administered within 6 weeks after surgery for patients with ENE or positive surgical margins unless the patients were ineligible (medically unfit or refusal of chemotherapy). During the study period, different RT techniques were applied. We used conventional methods (bilateral opposition with lower anterior neck portals, followed by a posterior electron boost to the neck) or three-dimensional conformal RT prior to 2001; intensity-modulated radiotherapy (IMRT) became the standard-of-care from 2001 onwards. The irradiation volume consisted of the primary tumor bed plus the neck lymphatic basin, either unilateral or bilateral, at the discretion of the radiation oncologist. ENE was not an absolute indication for using unilateral or bilateral RT in patients with BMSCC at our institution. For the uninvolved LN regions and all surgical beds, the patients received a prophylactic dose of 46–50 Gy, whereas for the primary tumor bed and involved LNs, they received an additional boost of 66 Gy with a 0.5- to 1- cm margin. The chemotherapy regimen included weekly cisplatin 40 mg/m^2^ or cisplatin 100 mg/m^2^ every 3 weeks [28,37].

### 4.3. Study Variables

The age of 40 years was selected as the cutoff according to prior studies on oral squamous cell carcinoma [38]. Likewise, margin status was classified into adequate (≥5 mm), close (<5 mm), and positive (<1 mm) margins based on previous studies [27,39]. RT treatment duration and surgery to RT interval were dichotomized using the cutoff values of 8 weeks [40] and 6 weeks [41], respectively. In terms of the optimal cutoff value for ENE number, receiver operating characteristic curve analysis was applied. The value was then tested using the Kaplan–Meier method based on the 3-year contralateral nodal control (CLNC) rate. The cutoff point of ENE of four nodes (≥4 nodes versus <4 nodes) yielded the highest significance regarding CLNC.

### 4.4. Outcomes

The primary outcome was CLNC. The secondary outcomes were overall survival (OS), disease-free survival (DFS), distant metastasis-free survival (DMFS), regional control (RC), and local control (LC). All the outcomes were calculated from the date of surgery to the date of the event of interest (or data were censored on the date of the last follow-up).

### 4.5. Propensity Score Matching

To control the imbalance of critical clinicopathological factors between treatment groups, we utilized the propensity score matching (PSM) method [42]. Propensity scores were estimated using the logistic regression model for each study patient based on the predetermined clinical, pathological, and treatment variables. Clinical variables included age, sex, performance status (Eastern Cooperative Oncology Group 0–1 or ≥2), Charlson comorbidity index (0–2 or ≥3), smoking status (nonsmoker or current/ex-smoker), alcohol drinking (non-drinker or current/ex-drinker), and betel quid chewing (non-chewer or current/ex-chewer). Pathological variables included pathologic tumor stage (pT1–2 or pT3–4), nodal stage (pN2a or pN3b), differentiation (well–moderate or poor), margin status (adequate or close/positive), ENE number, lymphatic invasion, vascular invasion, perineural invasion, soft tissue invasion, bone invasion, and skin invasion. Treatment variables included the use of concurrent chemotherapy, RT treatment duration (<8 wk or ≥8 wk), and surgery to RT interval (<6 wk or ≥6 wk). Subsequently, a 1:2 match between the unilateral and bilateral RT arm was performed using nearest neighbor matching without replacement, with a caliper distance of 25% of the standard deviation of the propensity scores.

### 4.6. Statistical Analysis

Dichotomous and continuous variables were compared using the chi-square test (or Fisher’s exact test) and Student’s t-test (or Mann–Whitney *U* test), respectively. Survival curves were estimated using the Kaplan–Meier method, and significance was evaluated using log-rank tests. Univariate and multivariate hazard ratios were calculated using a Cox proportional hazard model. In addition to RT laterality (unilateral vs. bilateral RT), all prognostic factors showing significant associations *(p* < 0.05) in univariate analysis were included in the multivariate model. Multicollinearity between variables was examined based on the variance inflation factor. The prognostic model for CLNC was built based on results of the decision tree analysis using Chi-square automatic detection [43,44]. This method dichotomized the whole dataset with the most significant risk factor (with the largest chi-square), which continued in a stepwise fashion to select the next most influential risk factor. Risk groups were then refined according to survival curves. All statistical tests were two-sided, and a *p*-value of <0.05 was considered statistically significant. Analyses were performed using IBM SPSS statistical software (version 21; SPSS, Inc., Chicago, IL, USA).

## 5. Conclusions

This study showed that postoperative unilateral RT is a safe treatment modality for patients with ENE+ BMSCC. Mostly, contralateral nodal failure occurred concomitantly or was preceded by distant failure. Our data also demonstrated that an ENE number ≥4 was an independent risk factor for CLNC. The proposed prognostic model may provide insight into the incidence of contralateral nodal failure and help clinicians tailor radiation fields but also consider more aggressive adjuvant systemic therapy.

## Figures and Tables

**Table 1:** Comparison of clinicopathological characteristics before and after propensity score matching.

	Unmatched Groups			Propensity Score-Matched Groups		
Characteristic	Unilateral RT (*n* = 61)	Bilateral RT (*n* = 125)	*p* Value	Unilateral RT (*n* = 45)	Bilateral RT (*n* = 78)	*p* Value
	*n* (%)	*n* (%)		*n* (%)	*n* (%)	
Treatment time periods	1999–2016	1997–2016		1999–2016	1997–2016	
Age, years, mean ± SD	48.9 ± 11.66	50.5 ± 9.67	0.316	50.3 ± 11.05	50.0 ± 9.55	0.871
<40	11 (18.0)	17 (13.6)	0.427	5 (11.1)	11 (14.1)	0.635
≥40	50 (82.0)	108 (86.4)		40 (88.9)	67 (85.9)	
Sex, male	57 (93.4)	122 (97.6)	0.219	44 (97.8)	75 (96.2)	1.000
Smoking status						
No	12 (19.7)	28 (22.4)	0.671	8 (17.8)	13 (16.7)	0.875
Yes	49 (80.3)	97 (77.6)		37 (82.2)	65 (83.3)	
Betel quid chewing						
No	13 (21.3)	31 (24.8)	0.599	9 (20.0)	16 (20.5)	0.946
Yes	48 (78.7)	94 (75.2)		36 (80.0)	62 (79.5)	
Alcohol drinking						
No	15 (24.6)	32 (25.6)	0.882	10 (22.2)	16 (20.5)	0.823
Yes	46 (75.4)	93 (74.4)		35 (77.8)	62 (79.5)	
ECOG, ≥2	1 (1.6)	3 (2.4)	1.000	1 (2.2)	1 (1.3)	1.000
CCI, ≥3	11 (18.0)	22 (17.6)	0.942	9 (20.0)	15 (19.2)	0.917
AJCC 8th pT classification ^a^						
T1–2	20 (32.8)	44 (35.2)	0.745	17 (37.8)	30 (38.5)	0.940
T3–4	41 (67.2)	81 (64.8)		28 (62.2)	48 (61.5)	
AJCC 8th pN classification ^a^						
N2a	12 (19.7)	25 (20.0)	0.958	8 (17.8)	15 (19.2)	0.842
N3b	49 (80.3)	100 (80.0)		37 (82.2)	63 (80.8)	
Differentiation						
Well–moderate	52 (85.2)	95 (76.0)	0.146	37 (82.2)	65 (83.3)	0.875
Poor	9 (14.8)	30 (24.0)		8 (17.8)	13 (16.7)	
Margin ^a^						
Adequate	45 (73.8)	86 (68.8)	0.831	33 (73.3)	56 (71.8)	1.000
Close	13 (21.3)	30 (24.0)		9 (20.0)	17 (21.8)	
Positive	3 (4.9)	9 (7.2)		3 (6.7)	5 (6.4)	
ENE number ^b^, mean ± SD	2.2 ± 1.88	2.2 ± 2.29	0.878	2.4 ± 1.98	2.1 ± 1.68	0.452
ENE number ^b^ ≥4	14 (23.3)	20 (16.4)	0.259	12 (26.7)	13 (17.1)	0.209
Lymphatic invasion, present	13 (21.3)	12 (9.6)	0.028 *	7 (15.6)	9 (11.5)	0.524
Vascular invasion, present	6 (9.8)	14 (11.2)	0.778	6 (13.3)	9 (11.5)	0.770
PNI, present	30 (49.2)	76 (60.8)	0.133	23 (51.1)	41 (52.6)	0.877
Soft tissue invasion, present	46 (75.4)	110 (88.0)	0.028 *	34 (75.6)	64 (82.1)	0.389
Bone invasion, present	22 (36.1)	39 (31.2)	0.507	17 (37.8)	26 (33.3)	0.619
Skin invasion, present	10 (16.4)	33 (26.4)	0.129	8 (17.8)	16 (20.5)	0.712
Chemotherapy (CDDP-based)	53 (86.9)	116 (93.5)	0.130	41 (91.1)	72 (93.5)	0.625
RT interval						
<8 wk	44 (72.1)	101 (80.8)	0.181	34 (75.6)	64 (82.1)	0.389
≥8 wk	17 (27.9)	24 (19.2)		11 (24.4)	14 (17.9)	
Surgery to RT interval						
<6 wk	32 (52.5)	79 (63.2)	0.161	23 (51.1)	52 (66.7)	0.088
≥6 wk	29 (47.5)	46 (36.8)		22 (48.9)	26 (33.3)	
Median RT dose, Gy (range)	66 (64.0–70.0)	66 (60.0–82.0)	0.466	66 (64.0–66.8)	66 (60.0–79.2)	0.905
RT technique						
2D-RT/3D-CRT	14 (23.0)	5 (4.0)	<0.001 *	8 (17.8)	5 (6.4)	0.048 *
IMRT/VMAT	47 (77.0)	120 (96.0)		37 (82.2)	73 (93.6)	

Abbreviations: CCI, Charlson comorbidity index; CDDP, cisplatin; CRT, conformal radiotherapy; ECOG, Eastern Cooperative Oncology Group performance; ENE, extranodal extension; IMRT, intensity-modulated radiotherapy; PNI, perineural invasion; RT, radiotherapy; SD, standard deviation; VMAT, volumetric arc therapy. ^a^ Adequate ≥ 5 mm, close < 5 mm, positive < 1 mm. ^b^ Data were not available for 4 patients. * *p* < 0.05 between the two groups for a given variable.

**Table 2:** Significant prognostic factors in multivariate analysis in the propensity-matched cohort.

Variable	Hazard Ratio (95% CI)	*p* Value ^a^
CLNC		
Unilateral RT vs. Bilateral RT	1.05 (0.33–3.28)	NS
IMRT/VMAT vs. 2D-RT/3D-CRT	**	NS
ENE number (≥4 vs. <4)	4.85 (1.50–15.74)	0.008 *
Close/positive margin	1.82 (0.57–5.79)	NS
OS		
Unilateral RT vs. Bilateral RT	1.09 (0.65–1.80)	NS
IMRT/VMAT vs. 2D-RT/3D-CRT	0.77 (0.31–1.95)	NS
Chemotherapy (CDDP-based)	0.55 (0.24–1.25)	NS
AJCC 8th T classification (pT3–4 vs. pT1–2)	1.90 (1.01–3.60)	0.048 *
ENE number (≥4 vs. <4)	2.24 (1.30–3.85)	0.004 *
Lymphatic invasion	1.99 (1.01–3.94)	0.047 *
PNI	1.40 (0.83–2.35)	NS
Bone invasion	0.92 (0.52–1.65)	NS
DFS		
Unilateral RT vs. Bilateral RT	1.04 (0.63–1.73)	NS
IMRT/VMAT vs. 2D-RT/3D-CRT	0.69 (0.29–1.66)	NS
AJCC 8th T classification (pT3–4 vs. pT1–2)	1.97 (1.05–3.71)	0.036 *
ENE number (≥4 vs. <4)	2.30 (1.34–3.94)	0.002 *
Lymphatic invasion	2.03 (1.03–4.01)	0.041 *
Bone invasion	1.06 (0.60–1.85)	NS
DMFS		
Unilateral RT vs. Bilateral RT	1.15 (0.58–2.30)	NS
IMRT/VMAT vs. 2D-RT/3D-CRT	**	NS
AJCC 8th T classification (pT3–4 vs. pT1–2)	2.41 (1.09–5.33)	0.030 *
ENE number (≥4 vs. <4)	2.35 (1.15–4.81)	0.019 *
LC		
Unilateral RT vs. Bilateral RT	0.43 (0.19–1.01)	NS
IMRT/VMAT vs. 2D-RT/3D-CRT	0.33 (1.00–1.09)	NS
Surgery to RT interval, wk (≥6)	3.17 (1.43–7.02)	0.004 *
AJCC 8th T classification (pT3–4 vs. pT1–2)	2.18 (0.91–5.20)	NS
ENE number (≥4 vs. <4)	4.04 (1.78–9.19)	0.001 *
RC		
Unilateral RT vs. Bilateral RT	0.65 (0.27–1.59)	NS
IMRT/VMAT vs. 2D-RT/3D-CRT	**	NS
AJCC 8th T classification (pT3–4 vs. pT1–2)	4.22 (1.15–15.48)	0.030 *
ENE number (≥4 vs. <4)	4.30 (1.90–9.73)	<0.001 *
Bone invasion	1.33 (0.56–3.15)	NS

Abbreviations: AJCC, American Joint Committee on Cancer; CDDP, cisplatin; CI, confidence interval; CLNC, contralateral nodal control; CRT, conformal radiotherapy; DFS, disease-free survival; DMFS, distant metastasis-free survival; ENE, extranodal extension; HR, hazard ratio; IMRT, intensity-modulated radiotherapy; LC, local control; NS, not statistically significant; OS, overall survival; PNI, perineural invasion; RC, regional control; RT, radiotherapy; VMAT, volumetric arc therapy. ^a^ RT laterality, RT techniques, and all factors with *p* < 0.05 in the univariate analysis were included in the multivariate Cox proportional hazard model. * *p* < 0.05 between the two groups for a given variable. ** Do not converge.

**Table 3:** Characteristics of patients with contralateral nodal failure.

No.	ENE Number	Pathological Margin Status ^a^	T-Stage	RT Laterality	Site of cNF	Coincided with or Preceded by			Salvage Treatment
						LR	iNF	DM	
1	<4	Adequate	pT3	Bilateral	I, II, III, RP node	●			Surgery + PORT + CT
2	<4	Adequate	pT4	Bilateral	I, IV, VI		●	●	
3	<4	Close/positive	pT4	Unilateral	IV			●	
4	<4	Adequate	pT4	Unilateral	IV			●	
5	<4	Adequate	pT3	Bilateral	I			●	
6	<4	Adequate	pT4	Bilateral	II	●			
7	≥4	Adequate	pT3	Unilateral	II				Surgery + PORT
8	≥4	Close/positive	pT2	Bilateral	I				Surgery + PORT
9	≥4	Close/positive	pT2	Bilateral	I			●	
10	≥4	Close/positive	pT3	Bilateral	V, RP node	●		●	
11	≥4	Close/positive	pT4	Bilateral	II			●	
12	≥4	Close/positive	pT4	Unilateral	I, II, III, IV, VI	●		●	
13	≥4	Close/positive	pT2	Unilateral	II	●		●	

Abbreviations: cNF, contralateral nodal failure; CT, chemotherapy; DM, distant metastasis; ENE, extranodal extension; iNF, ipsilateral nodal failure; LR, local recurrence; PORT, postoperative radiotherapy; RP, retropharyngeal; RT, radiotherapy. ^a^ Adequate ≥ 5 mm, close < 5 mm, positive < 1 mm. ● denotes the types of recurrence concomitant or antecedent to contralateral nodal failures.

**Table 4:** Acute and late complications of the patients treated with bilateral radiotherapy (RT) versus unilateral radiotherapy (RT) in the propensity-matched cohort.

Complication	Grade ^a^	Bilateral RT *n* (%)	Unilateral RT *n* (%)	*p* Value ^b^
Acute				
Xerostomia	<3	32/34 (94.1)	23/23 (100.0)	0.510
	≥3	2/34 (5.9)	0/23 (0.0)	
Oral mucositis	<3	37/70 (52.9)	23/39 (59.0)	0.538
	≥3	33/70 (47.1)	16/39 (41.0)	
Dermatitis	<3	50/54 (92.6)	18/19 (94.7)	1.000
	≥3	4/54 (7.4)	1/19 (5.3)	
Late				
Xerostomia	<2	23/30 (76.7)	20/28 (71.4)	0.649
	≥2	7/30 (23.3)	8/28 (28.6)	
Soft Tissue Fibrosis	<2	8/10 (80.0)	11/13 (84.6)	1.000
	≥2	2/10 (20.0)	2/13 (15.4)	

^a^ Radiation Therapy Oncology Group acute and late morbidity scoring criteria: ^b^ Chi-square or Fisher exact test.

## Data Availability

The data presented in this study are not publicly available for privacy and legal reasons.

## Supplementary Materials

The following are available online at https://www.mdpi.com/article/10.3390/cancers13235997/s1, Figure S1: Failure pattern among 62 patients with recurrent tumors, Figure S2: The Kaplan-Meier estimates of (**A**) contralateral nodal control (CLNC), (**B**) overall survival (OS), (**C**) disease-free survival (DFS), (**D**) distant metastasis-free survival (DMFS), (**E**) local control (LC), and (**F**) regional control (RC) for patients treated with bilateral radiotherapy (RT) (blue line) versus unilateral RT (red line) in the propensity-matched cohort (exclusion of patients not receiving adjuvant cisplatin-based concurrent chemoradiation), Figure S3: The Kaplan-Meier estimates of contralateral nodal control of (**A**) 76 patients receiving bilateral RT and (**B**) 45 patients receiving unilateral RT stratified by the proposed prognostic model in the propensity-matched cohort, Table S1: Nodal irradiated volumes, Table S2: Univariate analysis of prognostic factors in the propensity-matched cohort, Table S3: Elimination of contralateral neck irradiation treatment for oral and oropharyngeal cancer.
