# Supplementary material for: Efficacy of Postoperative Unilateral Neck Irradiation in Patients with Buccal Mucosa Squamous Carcinoma with Extranodal Extension: A Propensity Score Analysis
Source: Cancers (Basel). 2021 Nov 29;13(23):5997. doi: 10.3390/cancers13235997 (PMC8656711; doi:10.3390/cancers13235997)
Supplement: Supplementary file 1 [file cancers-13-05997-s001.zip › cancers-1386156-supplementary.pdf]

cancers

MDPI

Article

Supplementary: Efficacy of Postoperative Unilateral Neck Irradiation in Patients with Buccal Mucosa Squamous Carcinoma with Extranodal Extension: A Propensity Score Analysis

Chia-Hsin Lin, Chien-Yu Lin, Kang-Hsing Fan, Sheng-Ping Hung, Yung-Chih Chou, Chia-Jen Liu, Wen-Chi Chou, Yen-Chao Chen, Shiang-Fu Huang, Chung-Jan Kang, Kai-Ping Chang, Hung-Ming Wang, Ann-Joy Cheng and Joseph Tung-Chieh Chang

Table S1. Nodal irradiated volumes.

	Unmatched groups			Propensity score-matched groups		
**Nodal levels included**	**Unilateral RT (n = 61)**	**Bilateral RT (n = 125)**	***p* value**	**Unilateral RT (n = 45)**	**Bilateral RT (n = 78)**	***p* value**
	***n* (%)**	***n* (%)**		***n* (%)**	***n* (%)**	
Ipsilateral						
I–II	1 (1.6)	0 (0.0)	0.690	1 (2.2)	0 (0.0)	0.545
I–III	0 (0.0)	1 (0.8)		0 (0.0)	1 (1.3)	
I–V	7 (11.5)	10 (8.0)		6 (13.3)	6 (7.7)	
I–V + SCF	46 (75.4)	98 (78.4)		34 (75.6)	59 (75.6)	
I–V + RP	0 (0.0)	1 (0.8)		0 (0.0)	1 (1.3)	
I–V + SCF + RP	7 (11.5)	15 (12.0)		4 (8.9)	11 (14.1)	
Contralateral						
I	NA	2 (1.6)	NA	NA	2 (2.6)	NA
I–II	NA	61 (48.8)		NA	34 (43.6)	
I–III	NA	22 (17.6)		NA	16 (20.5)	
I–IV	NA	2 (1.6)		NA	1 (1.3)	
I–III + V	NA	1 (0.8)		NA	1 (1.3)	
I-V	NA	6 (4.8)		NA	3 (3.8)	
I–V + SCF	NA	19 (15.2)		NA	10 (12.8)	
I–V + RP	NA	1 (0.8)		NA	1 (1.3)	
I–V + SCF + RP	NA	11 (5.9)		NA	10 (12.8)	

Abbreviations: NA, not applicable; RP, retropharyngeal; RT, radiotherapy; SCF, supraclavicular fossa.

 Cancers **2021**, *13*, 5997

Table S2. Univariate analysis of prognostic factors in the propensity-matched cohort.

Variables	CLNC		OS		DFS		DMFS		LC		RC	
	HR (95% CI)	*p* value	HR (95% CI)	*p* value	HR (95% CI)	*p* value	HR (95% CI)	*p* value	HR (95% CI)	*p* value	HR (95% CI)	*p* value
Unilateral RT vs. Bilateral RT	1.20 (0.39–3.67)	0.749	1.34 (0.83–2.14)	0.231	1.16 (0.74–1.82)	0.515	1.26 (0.64–2.48)	0.500	0.86 (0.40–1.85)	0.692	0.72 (0.30–1.73)	0.467
RT interval, wk (≥ 8)	0.32 (0.04–2.47)	0.276	1.27 (0.74–2.17)	0.388	1.43 (0.87–2.36)	0.159	1.17 (0.53–2.58)	0.692	1.46 (0.65–3.30)	0.361	0.52 (0.16–1.75)	0.294
RT technique (IMRT/VMAT vs. 2D-RT/3D-CRT)	**	0.417	0.96 (0.46–2.00)	0.904	0.97 (0.48–1.93)	0.920	**	0.188	0.80 (0.28–2.31)	0.682	2.87 (0.39–21.20)	0.302
Surgery to RT interval, wk (≥ 6)	1.04 (0.34–3.19)	0.940	1.32 (0.83–2.10)	0.249	1.33 (0.85–2.06)	0.213	0.85 (0.42–1.71)	0.646	3.34 (1.56–7.14)	0.002*	1.11 (0.50–2.46)	0.805
Chemotherapy (CDDP-based)	0.74 (0.10–5.73)	0.777	0.43 (0.20–0.90)	0.024*	0.51 (0.24–1.06)	0.072	0.68 (0.21–2.21)	0.515	0.74 (0.18–3.15)	0.688	0.73 (0.17–3.09)	0.664
Age, years (≥ 40 vs. < 40)	0.85 (0.19–3.86)	0.836	0.87 (0.45–1.70)	0.686	0.94 (0.50–1.78)	0.845	1.21 (0.43–3.42)	0.723	0.95 (0.33–2.73)	0.922	1.16 (0.35–3.87)	0.811
Sex (male vs. female)	**	0.669	0.70 (0.22–2.23)	0.545	0.85 (0.27–2.68)	0.774	1.13 (0.15–8.24)	0.907	0.93 (0.13–6.87)	0.946	**	0.546
Smoking status (yes vs. no)	2.59 (0.34–19.89)	0.361	1.05 (0.57–1.92)	0.871	1.00 (0.58–1.73)	0.991	1.09 (0.45–2.64)	0.842	1.63 (0.56–4.70)	0.367	1.60 (0.48–5.33)	0.448
Betal quid chewing (yes vs. no)	**	0.266	0.76 (0.44–1.31)	0.318	0.81 (0.48–1.35)	0.413	0.83 (0.38–1.82)	0.639	0.82 (0.35–1.92)	0.649	1.30 (0.45–3.78)	0.633
Alcohol drinking (yes vs. no)	0.82 (0.23–2.99)	0.766	0.82 (0.47–1.44)	0.497	0.97 (0.57–1.65)	0.899	1.23 (0.51–2.97)	0.641	1.00 (0.41–2.45)	0.998	1.01 (0.38–2.70)	0.983
ECOG (≥ 2 vs. < 2)	**	0.738	1.48 (0.36–6.03)	0.588	1.24 (0.30–5.05)	0.766	1.47 (0.20–10.76)	0.704	**	0.616	2.18 (0.29–16.12)	0.446
CCI (≥ 3 vs. < 3)	0.82 (0.18–3.72)	0.800	1.62 (0.94–2.82)	0.085	1.53 (0.91–2.57)	0.109	1.59 (0.74–3.40)	0.232	1.52 (0.65–3.56)	0.340	1.40 (0.56–3.50)	0.477
AJCC 8th T classification (pT3–4 vs. pT1–2)	2.72 (0.75–9.95)	0.130	2.37 (1.40–4.02)	0.001*	2.32 (1.43–3.78)	0.001*	2.75 (1.24–6.08)	0.013*	2.36 (1.05–5.33)	0.038*	5.96 (1.78–19.96)	0.004*
AJCC 8th N classification (pN3b vs. pN2a)	3.39 (0.44–26.07)	0.241	1.84 (0.94–3.60)	0.076	1.82 (0.98–3.38)	0.057	2.14 (0.76–6.08)	0.152	2.11 (0.73–6.12)	0.171	6.77 (0.92–50.04)	0.061
Differentiation, poor	**	0.315	1.10 (0.59–2.04)	0.774	0.99 (0.55–1.79)	0.972	1.35 (0.59–3.08)	0.483	0.56 (0.17–1.85)	0.342	0.70 (0.21–2.35)	0.568

Cancers 2021, 13, 5997

Close/positive margin	3.04 (1.02–9.06)	0.045*	0.92 (0.54–1.55)	0.742	0.95 (0.58–1.56)	0.833	1.40 (0.69–2.80)	0.350	0.76 (0.32–1.78)	0.524	1.49 (0.66–3.37)	0.341
ENE number (≥ 4 vs. < 4)	6.42 (2.15–19.22)	0.001*	2.21 (1.32–3.69)	0.002*	2.78 (1.70–4.55)	< 0.001*	2.73 (1.36–5.50)	0.005*	3.06 (1.41–6.63)	0.005*	4.53 (2.02–10.14)	< 0.001*
Lymphatic invasion	**	0.485	2.39 (1.27–4.47)	0.007*	2.27 (1.25–4.13)	0.007*	1.39 (0.49–3.95)	0.542	2.27 (0.86–6.01)	0.098	0.95 (0.22–4.06)	0.947
Vascular invasion	1.48 (0.33–6.68)	0.613	1.25 (0.64–2.45)	0.508	1.09 (0.56–2.12)	0.798	0.95 (0.33–2.69)	0.918	0.86 (0.26–2.86)	0.811	1.11 (0.33–3.71)	0.866
PNI	1.29 (0.43–3.86)	0.645	1.88 (1.16–3.03)	0.010*	1.48 (0.95–2.30)	0.083	1.36 (0.70–2.65)	0.368	1.03 (0.50–2.12)	0.937	1.98 (0.87–4.48)	0.103
Soft tissue invasion	**	0.243	1.27 (0.70–2.33)	0.432	1.44 (0.80–2.56)	0.223	2.94 (0.90–9.62)	0.074	1.22 (0.49–3.03)	0.667	3.13 (0.74–13.26)	0.122
Bone invasion	1.37 (0.45–4.21)	0.578	1.66 (1.03–2.66)	0.036*	1.66 (1.06–2.59)	0.027*	1.51 (0.77–2.97)	0.236	1.09 (0.51–2.33)	0.824	2.37 (1.08–5.19)	0.032*
Skin invasion	1.64 (0.45–5.99)	0.452	1.54 (0.87–2.72)	0.142	1.53 (0.88–2.65)	0.132	1.67 (0.76–3.69)	0.205	1.69 (0.72–3.94)	0.229	1.71 (0.68–4.29)	0.253

Abbreviations: AJCC, American Joint Committee on Cancer; CCI, Charlson comorbidity index; CDDP, cisplatin; CI, confidence interval; CLNC, contralateral nodal control; CRT, conformal radiotherapy; ECOG, Eastern Cooperative Oncology Group performance; DFS, disease-free survival; DMFS, distant metastasis-free survival; ENE, extranodal extension; HR, hazard ratio; IMRT, intensity-modulated radiotherapy; LC, local control; OS, overall survival; PNI, perineural invasion; RC, regional control; RT, radiotherapy; VMAT, volumetric arc therapy. * $p < 0.05$ between the two groups for a given variable. **Don't converge

Table S3. Elimination of contralateral neck irradiation treatment for oral and oropharyngeal cancer.

Author	No. of patients	Site	N2/N3 *	T3/T4 *	ENE	cNF (overall)	cNF in ENE
Definitive RT							
Jackson et al [7]	178	Tonsil	13% (*n* = 23)	34% (*n* = 61)	NR	2.2% (*n* = 4)	NR
O' Sullivan et al [8]	228	Tonsil	17% (*n* = 39)	16% (*n* = 37)	NR	3.5% (*n* = 8)	NR
Liu et al [9]	58	Tonsil	33% (*n* = 19)	31% (*n* = 18)	NR	0%	NR
Murthy and Hendrickson [10]	32	Tonsil	31% (*n* = 10)	47% (*n* = 15)	NR	3.1% (*n* = 1)	NR
Kagei et al [11]	32	Tonsil<br>Soft palate	16% (*n* = 5)	43% (*n* = 14)	NR	0%	NR
Al-Mamgani et al [12]	185	Tonsil<br>Soft palate<br>Pharyngeal wall	27% (*n* = 50)	7% (*n* = 13)	7.8%	1.1% (*n* =2)	NR
Chronowski et al [13]	102	Tonsil	42% (*n* = 43)	NR	NR	2.0% (*n* = 2)	NR
Cramer et al [14]	23	Tonsil	96% (*n* = 22)	0%	NR	NR	NR
Kennedy et al [15]	76	Tonsil	45% (*n* = 34)	0%	NR	1.3% (*n* = 1)	NR
Hu et al [16]	37	Tonsil	65% (*n* = 24)	11% (*n* =4)	NR	0%	NR
Gottumukkala et al [17]	34	Tonsil	82% (*n* = 28)	9% (*n* = 3)	3% (n = 1)	2.9% (*n* = 1)	NR
Postoperative RT							
Cerezo et al [18]	20; definitive RT (*n* = 8) and postoperative RT (*n* = 12)	Tonsil<br>Oral cavity (tongue, retromolar, gum, and buccal mucosa)	25% (*n* = 5)	10% (*n* = 2)	NR	0%	NR
Hwang et al [19]	46	Tonsil	76% (*n* = 35)	NS	NR	0%	NR
Dan et al [20]	61; definitive RT (*n* = 17) and postoperative RT (*n* = 44)	Tonsil	75.4% (*n* = 46)	3% (*n* = 2)	28% (*n* = 17)	1.6% (*n* =1)	NR
Kim et al [21]	70 (after PSM)	Tonsil	63% (*n* = 44)	0%	31% (*n* = 22)	4.3% (*n* =3)	NR
Vergeer et al [22]	123	Tonsil<br>Base of tongue<br>Oral cavity (tongue, mouth floor, gum, and buccal mucosa)	22% (*n* = 27)	44% (*n* = 53)	20% (*n* = 25)	5.7% (*n* =7)	15% (5 year)
Lynch et al [23]	136	Tonsil	65% (*n* = 88)	4% (*n* = 5)	22% (*n* = 30)	5.9% (*n* =8)	20%
Chin et al [6]	48	Tonsil	79% (*n* = 38)	13% (*n* = 6)	77% (*n* = 37)	0%	0%
Contreras et al [24]	72	Oropharynx<br>Oral cavity<br>Hypopharynx<br>Larynx<br>Unknown primary	58% (*n* = 42)	51% (*n* = 37)	64% (*n* = 46)	2.8% (*n* =2)	NR
Current study	45 (after PSM)	Oral cavity (buccal mucosa)	82% (*n* = 37)	47% (*n* = 21)	100% (*n* = 45)	11.1% (*n* =5)	14.4% (3 year)

Abbreviations: cNF, contralateral nodal failure; ENE, extranodal extension; ND, neck dissection; NR, not reported; RT, radiotherapy; PSM, propensity score matching. * Based on the American Joint Committee on Cancer, 7th edition.

 Cancers **2021**, *13*, 5997

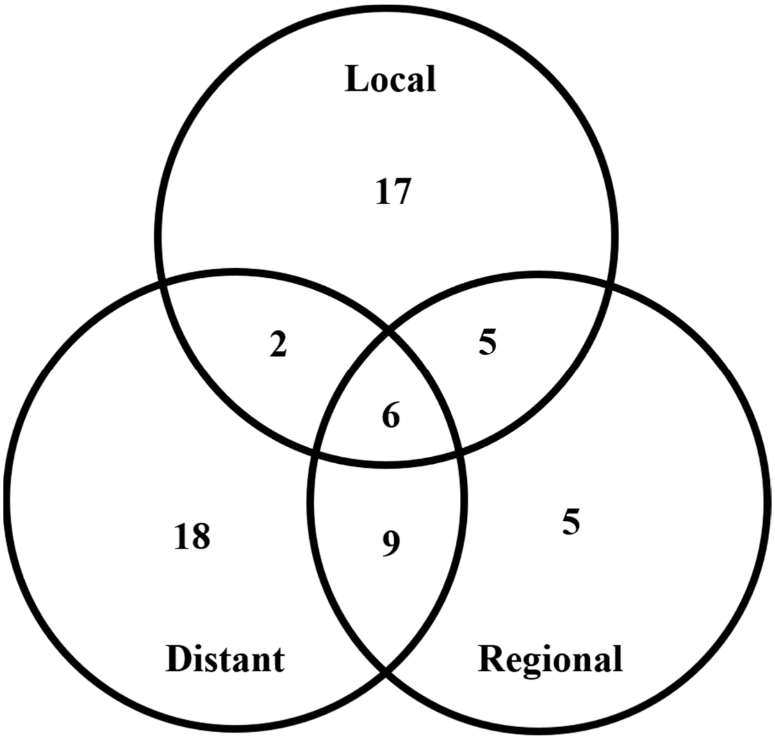

Figure S1. Failure pattern among 62 patients with recurrent tumors.

A

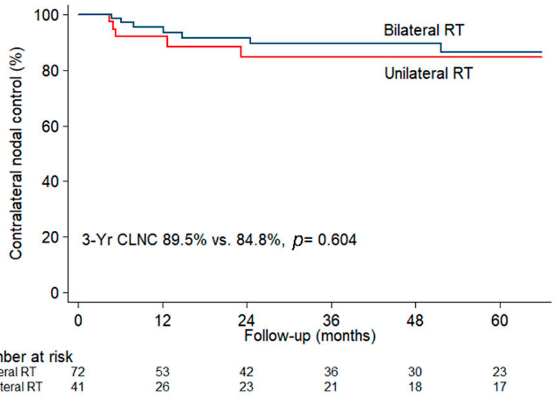

D

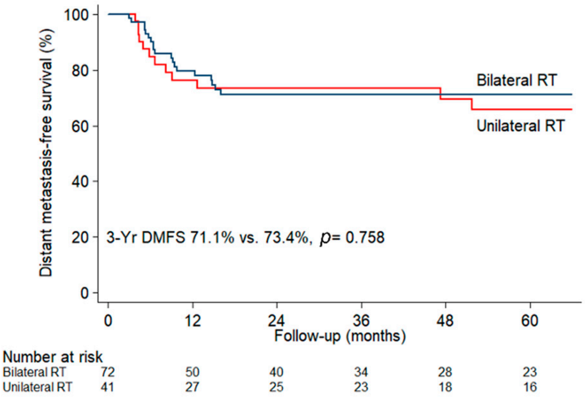

B

E

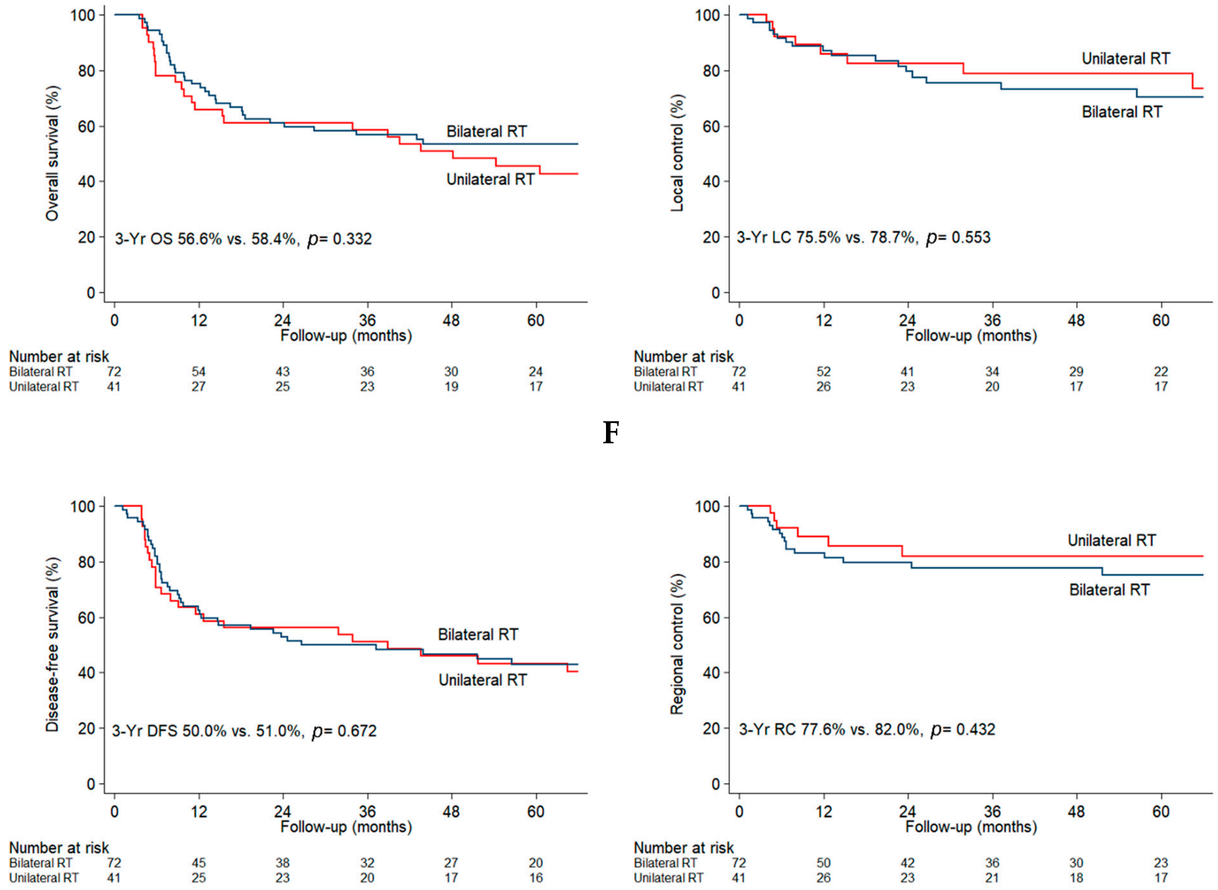

Figure S2. The Kaplan-Meier estimates of (**A**) contralateral nodal control (CLNC), (**B**) overall survival (OS), (**C**) disease-free survival (DFS), (**D**) distant metastasis-free survival (DMFS), (**E**) local control (LC), and (**F**) regional control (RC) for patients treated with bilateral radiotherapy (RT) (blue line) versus unilateral RT (red line) in the propensity-matched cohort (exclusion of patients not receiving adjuvant cisplatin-based concurrent chemoradiation).

A **B**

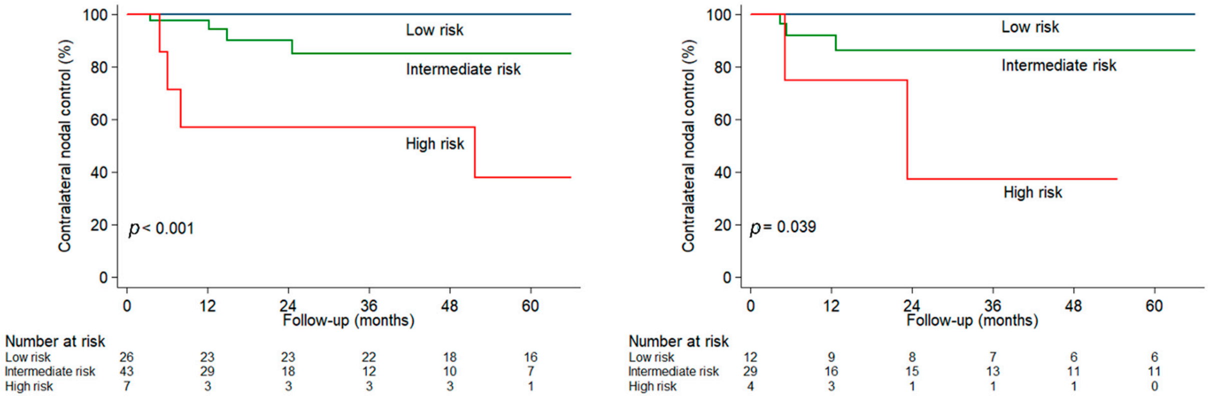

Figure S3. The Kaplan-Meier estimates of contralateral nodal control of (**A**) 76 patients receiving bilateral RT and (**B**) 45 patients receiving unilateral RT stratified by the proposed prognostic model in the propensity-matched cohort.
